# Unveiling unique allergenic properties of rapeseed oil: A clinical case study

**DOI:** 10.1016/j.jacig.2025.100493

**Published:** 2025-05-08

**Authors:** Margarida Gomes, Maria José Martinez, Fernando Pineda, Ana Mendes

**Affiliations:** a Serviço de Imunoalergologia, Hospital de Santa Maria, Unidade Local de Saúde Santa Maria, Lisboa, Portugal; b Inmunotek SL, Alcalá de Henares, Spain

**Keywords:** Food allergy, rapeseed, rapeseed oil

## Abstract

Rapeseed oil (*Brassica napus*), a plant from the Brassicaceae family, is widely utilized in culinary practices and industrial applications. Despite the prevalence of rapeseed oil, its allergenic potential remains largely unexplored, particularly in the context of food allergies. This study investigates a potential food allergy linked to rapeseed oil.

Rapeseed (*Brassica napus*), a plant from the Brassicaceae family, is a widely used source of oil in both industrial applications (eg, lubricants) and food products, particularly in Europe. Despite rapeseed’s widespread use, allergic reactions associated with it have been largely underreported. However, occupational exposure to rapeseed dust is associated with a significant risk of IgE-mediated bronchoconstriction among grain industry workers.^1^

The allopolyploid evolution of *Brassica napus*, originating from hybridization between *Brassica rapa* and *Brassica oleracea*, underlines its genetic complexity and adaptation. This genetic diversity is pivotal in elucidating the allergenic potential associated specifically with *Brassica napus* and its derivatives. The evolutionary trajectory of the *Brassica napus* genome following Neolithic agricultural practices underscores its historical and contemporary agricultural importance.^2^

A study conducted in India investigated hypersensitivity reactions to pollen extracts from *Brassica napus.* The findings revealed varying degrees of IgE-mediated hypersensitivity among atopic individuals. Major IgE-binding allergenic proteins identified from *Brassica napus* included those with molecular weights of 56, 76, 87, and 90 kDa. These findings manifest the necessity of comprehending local allergenic profiles for effective diagnosis and management of *Brassica napus*–related allergies and derivatives within the Indian population.^3^

The objective of this study was to investigate potential food allergy related to rapeseed oil. Informed consent was obtained.

## Methods

A 21-year-old male patient who reported cervical angioedema after ingesting biscuits, cheese snacks, and corn chips, all of which contained rapeseed oil. The patient reported no previous known allergy to these foods, but he had a history of allergic rhinitis in response to house dust mites.

Skin testing using samples of the biscuits, cheese snacks, and corn chips, as well as histamine, yielded positive results in the form of papule diameters of 6 × 5 mm for biscuits, 5 × 5 mm for cheese snacks and corn chips, and 2 × 2 mm for histamine. Rapeseed oil was identified as the only common ingredient among these foods.

A skin prick test using rapeseed oil was performed. The patient tested positive (papule diameter 6 × 5 mm). Immediately after the prick test using the rapeseed oil, the patient also developed an erythematous patch in the malar region, which was suggestive of urticaria. The results of testing of 2 controls were negative.

To delve deeper into the allergenic potential of rapeseed oil, various methods, namely, SDS-PAGE and Western blot, were used to determine its protein and allergenic profile.

**Fig 1:**
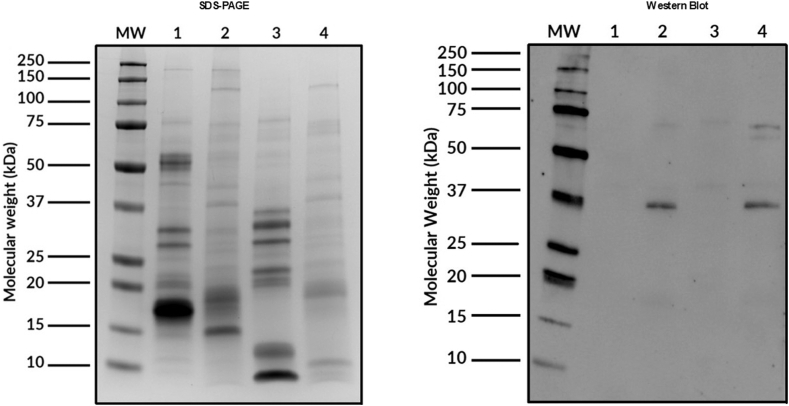
SDS-PAGE/IgE-Western blot in reducing and nonreducing conditions. (*Lanes 1 and 3*) Rapeseed, water-soluble part. (*Lanes 2 and 4*) Rapeseed, lipid-soluble part. Lanes 1 and 2 show nonreducing conditions; lanes 3 and 4 reducing show conditions.

The quantities of protein in the selected samples are presented in Table I. Regarding the Western blot results, 2 distinct protein bands (approximate molecular weights of 37 kDa and 70 kDa) were observed in the lipid portion of the extract under reducing and nonreducing conditions, respectively (Fig 1).

**Table I:** Protein quantity of selected rapeseed samples

Allergen	Protein concentration (mg/mL)
Rapeseed, water-soluble part	12.38
Rapeseed, lipid-soluble part	26.16

## Discussion and conclusions

After the patient's clinical history had been studied, a molecular analysis of the protein and allergen profiles of the samples was carried out. Western blot assay detected the presence of different protein bands in the lipid part of the rapeseed extract in both reducing and nonreducing conditions (at molecular weights of ∼37 kDa and ∼70 kDa, respectively). There are no rapeseed allergens with these characteristics described in the literature. It should be noted that the allergens to which our patient is sensitized are found only in the lipid part of the study food, coinciding with the patient’s clinical history, as his reactions were triggered by rapeseed oil.

This case study underscores the potential allergenicity of rapeseed oil and suggests the presence within its lipid portion of distinct protein bands that may be responsible for the patient's allergic responses, particularly in the context of the modern diet, in which rapeseed oil is increasingly being used in food products. The processing of canola oil makes it less allergenic than rapeseed oil, as it contains fewer proteins despite both oils having been derived from the same plant. The implications of rapeseed oil allergy are significant, as this oil is widely used in processed foods, restaurant cooking, and even nonfood products such as cosmetics and pharmaceuticals. Patients with confirmed sensitivity may need to adopt strict dietary vigilance and avoid products containing rapeseed oil. Additionally, improved food labeling and allergen testing are necessary to support individuals at risk. Clinicians should consider rapeseed oil as a potential hidden allergen in cases of unexplained allergic reactions.

The aforesaid findings call attention to the importance of further characterizing allergens to enhance the diagnosis and management of food allergies.

## Disclosure statement

Supported by Inmunotek.

Disclosure of potential conflict of interest: M. J. Martinez and F. Pineda are employed by Inmunotek. The rest of the authors declare that they have no relevant conflicts of interest.
